# Determination of oxygen saturation compared to a prescribed target range using continuous pulse oximetry in acutely unwell medical patients

**DOI:** 10.1186/s12890-021-01700-6

**Published:** 2021-10-26

**Authors:** James C. P. Harper, Ruth Semprini, Nethmi A. Kearns, Lee Hatter, Grace E. Bird, Irene Braithwaite, Allie Eathorne, Mark Weatherall, Richard Beasley

**Affiliations:** 1 Medical Research Institute of New Zealand, Private Bag 7902, Newtown, Wellington, 6242 New Zealand; 2 Victoria University, Wellington, New Zealand; 3 University of Otago Wellington, Wellington, New Zealand; 4 Capital & Coast District Health Board, Wellington, New Zealand; 5 Hutt Valley District Health Board, Wellington, New Zealand

**Keywords:** Oxygen saturation, Target range

## Abstract

**Background:**

Both inadequate and excessive administration of oxygen to acutely unwell patients results in risk of harm. Guidelines recommend titration of oxygen to achieve a target oxygen saturation (SpO_2_) range. Information regarding whether this is being achieved is limited.

**Methods:**

In this two-centre non-interventional study we used continuous pulse oximetry in acutely unwell medical patients over a 24-h period to determine the proportion of time spent with SpO_2_ within the prescribed target range and whether this is influenced by the target range, age, care in a high-dependency area and the number of oxygen adjustments.

**Results:**

Eighty participants were included in the analysis. The mean (SD) proportion of time spent in target range was 55.6% (23.6), this was lower in those with a reduced hypercapnic target range (88–92% or below) compared to those with a range of 92–96%; difference − 13.1% (95% CI − 3.0 to − 23.2), *P* = 0.012. The proportion of time spent above range was 16.2% (22.9); this was higher in those with a reduced hypercapnic range; difference 21.6% (31.4 to 12), *P* < 0.001. The proportion of time below range was 28.4% (25.2); there was no difference between target ranges. The proportion of time spent in range was higher for those in a high dependency area in the multivariate model; difference 15.5% (95% CI 2.3 to 28.7), *P* = 0.02.

**Conclusions:**

Medical patients receiving oxygen in a ward setting spend significant periods of time with SpO_2_ both above and below the prescribed target range while receiving oxygen therapy.

## Background

Oxygen is a commonly administered drug in hospital [1]. Despite the therapeutic use of oxygen for over 100 years [2], to our knowledge the first guideline to be published addressing the use of oxygen in the acute care setting was the 2008 British Thoracic Society (BTS) guideline for emergency oxygen use in adults [3]. This guideline was the first to clearly state that oxygen should be administered as a treatment for hypoxaemia and should not be administered indiscriminately to all acutely unwell patients or as a universal treatment for breathlessness. Specifically, the most recent BTS guideline [4] recommends the titration of oxygen to achieve a target peripheral oxygen saturation of 94–98%, with a lower target saturation range of 88–92% in patients at risk of hypercapnic respiratory failure [4]. The Thoracic Society of Australia and New Zealand (TSANZ) recommend a target range of 92–96% in patients without risk factors for hypercapnic respiratory failure, and the same 88–92% range in patients at risk of hypercapnic respiratory failure [5, 6]. These recommendations are supported by evidence for harm relating to the administration of excessive concentrations of oxygen in acute illnesses [7, 8], particularly in patients at risk of hypercapnic respiratory failure [9], as well as the known association between hypoxaemia and mortality [10, 11].

Despite strong evidence to support the avoidance of over and under-oxygenation, detailed information to determine whether this is being achieved in current clinical practice is limited. The 2015 BTS national emergency oxygen audit demonstrated only 69% of patients receiving oxygen had SpO_2_ within a prescribed range when evaluated at a single timepoint [12] and a 2017 Australian audit demonstrated SpO_2_ matched a prescribed range in 61% of patients admitted to an Acute Medical Unit [13]. However, these studies were limited by either single timepoint evaluation of SpO_2_ or evaluation according to intermittent SpO_2_ measurements by nursing staff. To our knowledge, there are no studies using continuous SpO_2_ monitoring, investigating the proportion of time patients spend with SpO_2_ within a prescribed target range and the time-exposure to oxygen saturation above and below range. This is important in order to understand the risk associated with oxygen therapy in current clinical practice and how this risk can be reduced.

We therefore conducted a non-interventional two-centre study using continuous oximetry for a period of 24 h to determine SpO_2_ compared to a prescribed target range in acutely unwell medical patients, and investigated which factors influenced the time spent in target range. Our hypothesis was that care in a high-dependency area, more frequent adjustments to oxygen delivery and prescription of a reduced hypercapnic target range would be associated with a greater proportion of time in range.

## Methods

### Study subjects

Wellington Regional Hospital (WRH) is a tertiary referral centre and teaching hospital in New Zealand with approximately 500 beds and Hutt Valley Hospital (HVH) is a regional hospital in New Zealand with approximately 320 beds. Adults admitted to WRH and HVH with an acute illness who were under the care of a medical team in a general ward or high dependency setting and were receiving oxygen with a clinician prescribed target SpO_2_ range were eligible for inclusion. Patients who were unable to consent, who had suspected or proven infection with COVID-19 or in whom continuous oximetry was not feasible were excluded. Patients with an expected duration of hospital admission of less than 24 h were also excluded.

### Study design

This was a two-centre non-interventional study. The study was approved by the Victoria University of Wellington Human Ethics Committee and was registered with the Australian and New Zealand Clinical Trials Registry (ACTRN12620000728932). The trial was run in accordance with Good Clinical Practice guidelines and the declaration of Helsinki.

### Methods

Consecutive patients under the care of a medical team who were receiving oxygen were screened by a study investigator. Potentially suitable patients were provided with an information sheet and written informed consent was obtained. Baseline information was collected including the primary reason for oxygen administration, whether there was a documented diagnosis of chronic obstructive pulmonary disease (COPD), obstructive sleep apnoea (OSA)/obesity hypoventilation syndrome (OHS), prescribed target SpO_2_ range, method of oxygen delivery and delivered oxygen flow or concentration at the time of enrolment. A disposable adhesive Masimo RD SET® Adt sensor (Masimo Corporation, Irvine, CA) was applied to a finger and connected to a small portable pulse oximeter (sat 801 + , Bitmos Düsseldorf, Germany). All pulse oximeter alarms were silenced and the screen was concealed to avoid influencing clinical management. The participant and nursing staff were advised that the sensor should be disconnected while mobilising, but at all other times should remain connected. Nursing staff were instructed to measure SpO_2_ using their normal ward pulse oximeter (VS-900, Mindray, Shenzhen, China) and adjust oxygen according to their normal practice. Physical observations, including SpO_2_, were measured by the nursing staff at a frequency determined by the patient’s early warning score (EWS) which at a minimum was six-hourly. All clinical care continued as normal during the study period. Participants were withdrawn from the study if admission to the intensive care unit (ICU) was required. After 24 h a study investigator removed the finger sensor and disconnected the pulse oximeter. The number of changes to the delivered oxygen flow or concentration, as well as periods when oxygen was stopped were recorded from the inpatient observation chart. Data was downloaded from the pulse oximeter using satView software (V1.1.9 Bitmos Düsseldorf, Germany). The pulse oximeter recorded SpO_2_, heart rate and signal quality every second, resulting in 86,400 measurements for each participant during the 24-h study period.

The primary objective of the study was to determine the proportion of time spent with SpO_2_ within the prescribed target range. Secondary objectives were to determine which factors influence the proportion of time spent in range, above range and below range as well the proportion of time spent with incremental deviation from the target range. We also investigated whether a greater proportion of time was spent in target range during the day (07:00 to 23:00) or night (23:00 to 07:00).

### Analysis

Analysis was undertaken per-protocol, whereby participants with at least six hours of SpO_2_ data recording with adequate signal quality (defined as > 50 on a scale of 0 to 100) while receiving oxygen were included. This was chosen to avoid potential bias arising from participants receiving oxygen for a short period of time and to reduce the chance of including spurious SpO_2_ values which may have arisen from movement artefact. We categorised the prescribed target ranges as 92–96%, as per TSANZ guidelines [6], and as reduced hypercapnic range for participants with a target range of 88–92% or a non-standard target range which was lower than 88–92%. An initial sample size of 100 was considered suitable to provide sufficient information, however due to uncertainty regarding the likely distribution of data, a pre-specified sample size analysis was undertaken after 25 participants. This determined a revised sample size of 80 would be sufficient to allow analysis of the effect of predictor covariates of age, prescribed SpO_2_ target range, care in a high-dependency area and number of oxygen adjustments on the proportion of time spent in target range.

SpO_2_ was rounded to an integer for all analyses. Proportion of time in range was calculated by dividing the number of SpO_2_ measurements within range by the total number of measurements. ANCOVA was used to estimate the strength of relationship between response variables and potential predictors. In the univariate models this was equivalent to simple linear regression for continuous variables and t-tests for categorical variables. In the multivariate models, all variables from the univariate models were incorporated. A paired t-test was used to compare the proportion of time spent in range for day versus night. For the density plots, nonparametric kernel density estimates with a standardisation bandwidth of 7 were overlaid on transparent histograms. For the large data sets of one measurement per second per participant over 24 h, every fifth measurement was used to manage the algorithm to generate the plots. SAS version 9.4 (SAS Institute, Cary, North Carolina) was used.

## Results

**Fig. 1:**
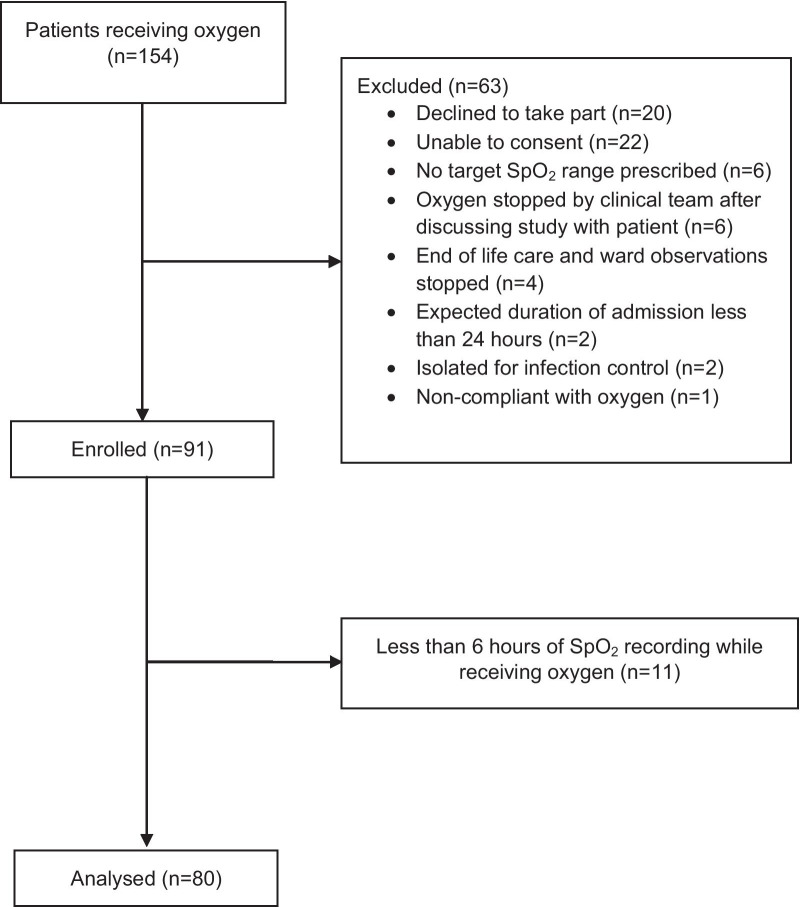
Study flow diagram

Participants were recruited to the study between 22^nd^ July 2020 and 30^th^ October 2020. 155 patients receiving oxygen were identified, and 91 were enrolled in the study. One patient died during the study period. For 11 participants, less than six hours of SpO_2_ data with adequate signal quality was recorded while receiving oxygen, leaving 80 participants included in the analysis: Fig. 1. The mean (SD) duration of SpO_2_ recording for participants included in the analysis was 18 (5.5) hours and the mean (SD) proportion of SpO_2_ data with adequate signal quality was 91.7% (9.6). Baseline participant characteristics are shown in Table 1 and prescribed target SpO_2_ ranges and methods of oxygen delivery at enrolment are shown in Table 2. The primary reasons for oxygen administration were heart failure and pneumonia which was present in 27 and 19 patients respectively. An acute exacerbation of COPD was present in 8 participants, however a total of 28 participants (35%) had a documented diagnosis of COPD. 17 participants (21.3%) had a documented diagnosis of OSA/OHS.

**Table 1:** Baseline participant characteristics

Characteristic N = 80	Mean (SD)
Age (years)	72.2 (12.7)
**Characteristic**	**N/80 (%)**
Female sex	39 (48.8)
**Ethnicity**	**N/80 (%)**
European	59 (73.8)
Pacific Peoples	10 (12.5)
Māori	7 (8.8)
Other	1 (1.3)
**Primary reason for oxygen requirement**	**N/80 (%)**
Heart failure	27 (33.8)
Pneumonia	19 (23.8)
Acute exacerbation of COPD	8 (10)
Pulmonary embolism	6 (7.5)
Pulmonary hypertension	4 (5.0)
Acute coronary syndrome	3 (3.8)
Interstitial lung disease	2 (2.5)
Lung cancer	2 (2.5)
Pleural effusion	2 (2.5)
Renal failure	2 (2.5)
Sepsis	2 (2.5)
Obesity hypoventilation syndrome	1 (1.3)
Pneumothorax	1 (1.3)
Atelectasis	1 (1.3)
**High dependency area**	**N/80 (%)**
Yes	20 (25)
No	60 (75)

**Table 2:** Oxygen delivery and prescribed target range at enrolment

Characteristic	
**Target SpO**_**2**_** range**	**N/80 (%)**
92–96%	41 (51.3)
Reduced hypercapnic^a^	34 (42.5)
Non-standard	5 (6.3)
**Method of oxygen delivery at enrolment**	**N/80 (%)**
Simple nasal prongs	64 (80)
Nasal high-flow	16 (20)
**Delivered oxygen at enrolment**	**Mean (SD)**
Simple nasal prongs (L/min)	1.6 (0.8)
Nasal high-flow (%)	32.3 (6.3)
Nasal high-flow (L/min)	36.5 (3.7)

^a^This group comprised the 28 participants with a target range of 88–92% and six patients with a non-standard target range which was lower than 88–92%. These ranges were 85–90%, 86–92%, 85–92%, 80–92% and two participants with 80–85%

There were 41 (51.3%) participants who were prescribed a standard target range of 92 to 96%, 34 (42.5%) a reduced hypercapnic range of 88 to 92% or lower, and five prescribed a non-standard range which could not be placed in either of these categories. Oxygen was administered by simple nasal prongs in 64 (80%) and by nasal high-flow in 16 (20%). One quarter of patients were admitted to a high dependency area during the period of the study.

**Fig. 2:**
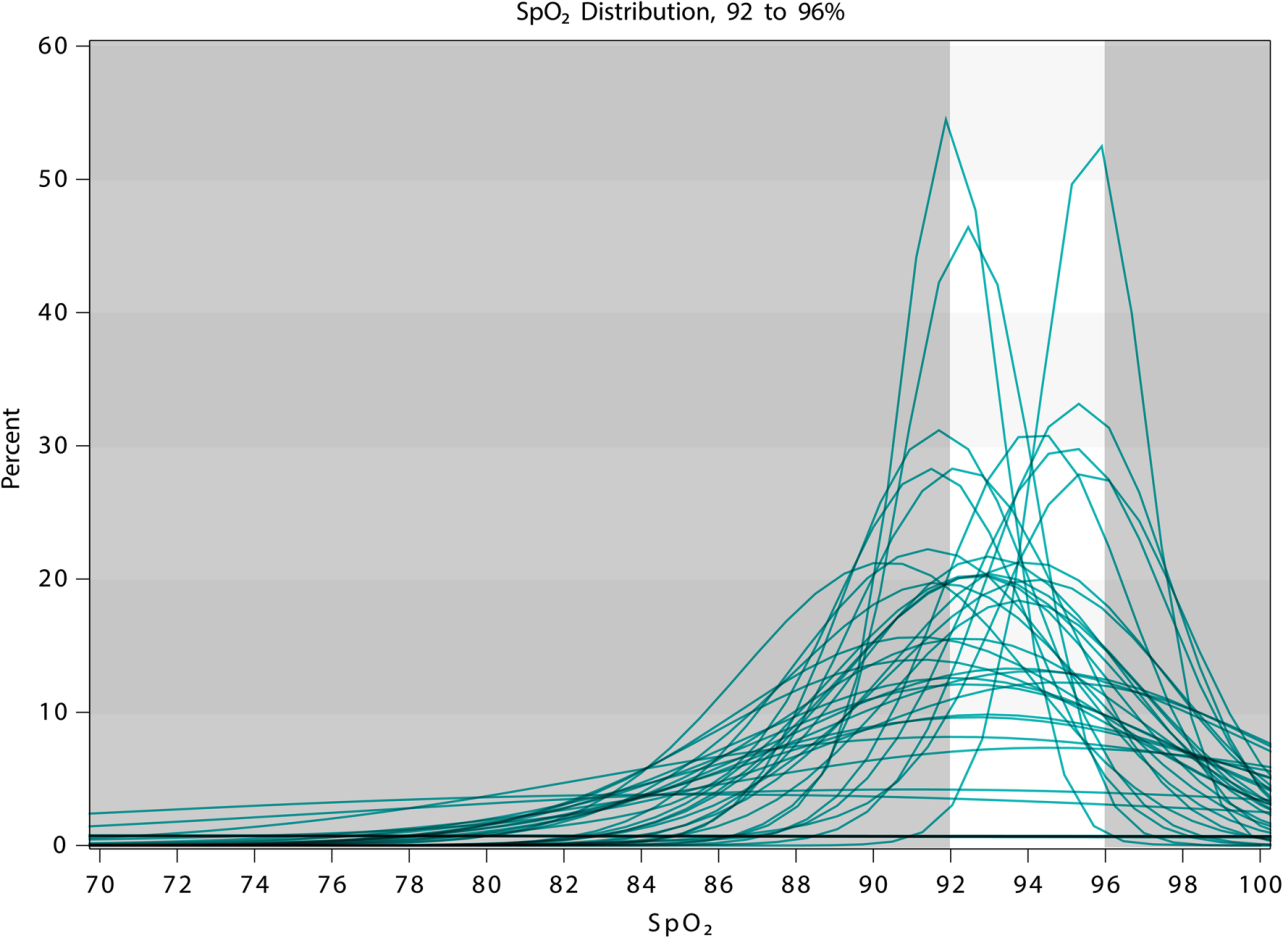
Histogram density plot for target SpO_2_ range 92–96%. Each line represents the distribution of SpO_2_ measurements for individual participants throughout the study period. The lightly shaded area represents the target range

**Fig. 3:**
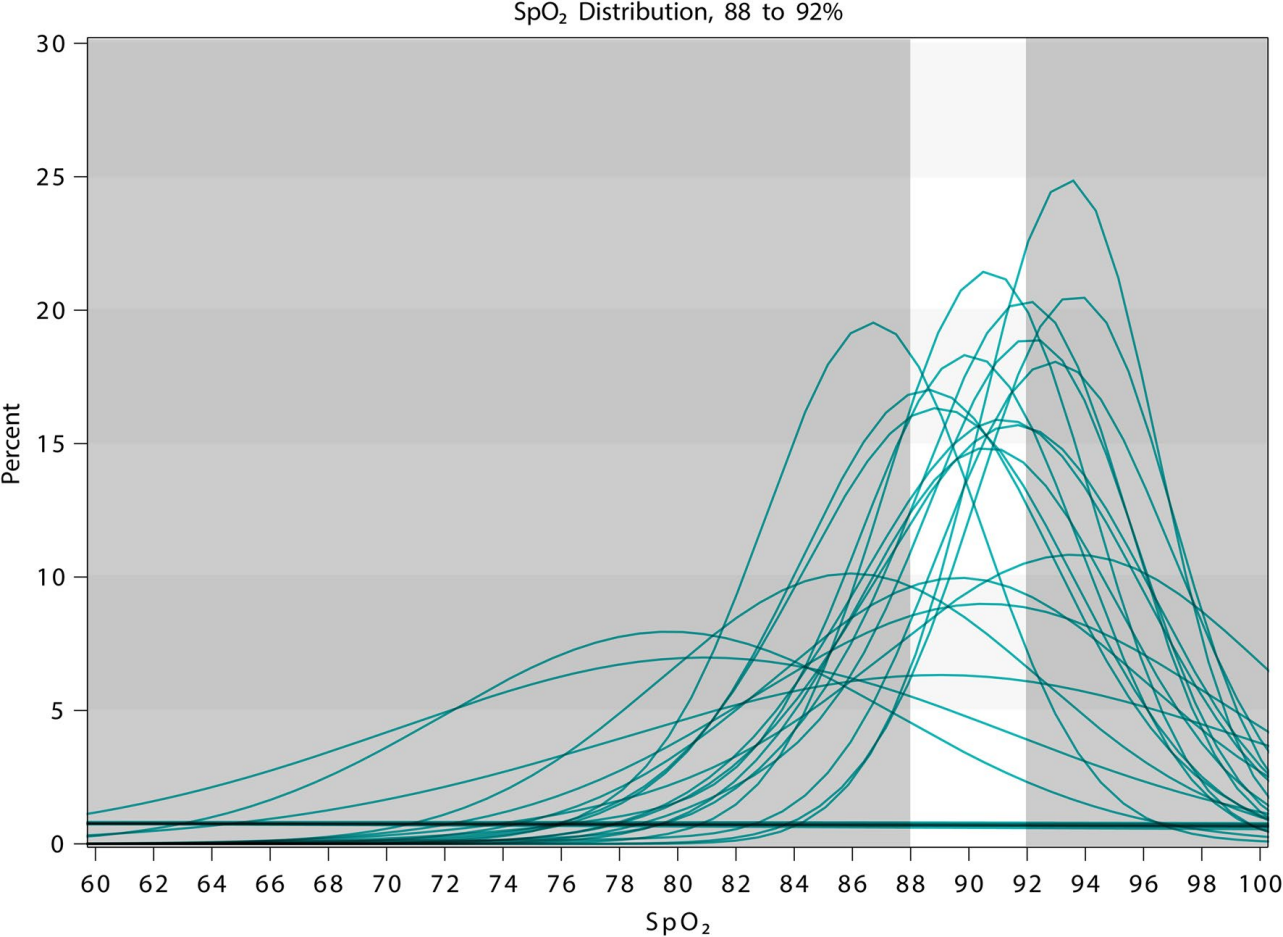
Histogram density plot for target SpO_2_ range 88–92%. Each line represents the distribution of SpO_2_ measurements for individual participants throughout the study period. The lightly shaded area represents the target range

The overall mean (SD) percentage of time spent with SpO_2_ in target range was 55.6% (23.6). The percentage of time spent above and below target range was 16.2% (22.9) and 28.4% (25.2) respectively. The percentage of time spent with progressive deviation from the prescribed target range is shown in Table 3. The percentage of time spent with SpO_2_ < 85% and < 80% in those with a reduced hypercapnic rage was 15% (22.5) and 5.2% (11) respectively, and in those with a target range of 92–96% was 2.1% (5.8) and 0.6% (2.6) respectively. Histogram density plots showing the distribution of SpO_2_ measurements for individual participants with prescribed ranges of 92–96% and 88–92% are shown in Figs. 2 and 3. Participants with non-standard ranges were omitted from these analyses.

**Table 3:** Percentage of time spent within, above and below target range

Percentage of time	All N = 80	Target 92–96% N = 41	Reduced hypercapnic N = 34
**Within range**	55.6 (23.6)	61.1 (19)	48.3 (25.2)
**Below range**	28.4 (25.2)	31.2 (22)	23.4 (26.5)
1–2% below	14.2 (11.7)	17.9 (12)	8.8 (8.3)
3–4% below	6.8 (8)	7.6 (7.1)	4.9 (6.4)
5–6% below	3 (4.3)	2.9 (3.9)	3.1 (4.7)
≥ 7% below	4.4 (11)	2.8 (6.8)	6.6 (15)
**Above range**	16.2 (22.9)	7.7 (13.3)	28.6 (27.6)
1–2% above	10.3 (12.7)	7 (11.5)	15.7 (13)
3–4% above	3.9 (8.5)	0.7 (2.4)	8.4 (11.4)
5–6% above	3.3 (8.8)^a^	NA	3.4 (8.9)
≥ 7% above	1.2 (4.2)^b^	NA	1.2 (4.2)

All values are mean (SD)

^a^N = 35 for participants with an upper SpO_2_ target range limit of < 96%

^b^N = 34 for participants with an upper SpO_2_ target range limit of < 94%

The analysis of predictor covariates is shown in Table 4. A reduced hypercapnic range was associated with a lower proportion of time spent in range compared to a target range of 92–96% in both univariate and multivariate models; multivariate difference (95% CI) − 13.1% (− 3 to − 23.2), *P* = 0.012. A reduced hypercapnic range was also associated with a greater proportion of time spent above range compared to a target range of 92–96% in both univariate and multivariate models; multivariate difference 21.6% (31.4 to 12), *P* < 0.001. There was no association between the prescribed range and proportion of time spent below range. There was an association between being cared for in a high dependency area and the proportion of time spent in range in the multivariate model only; difference 15.5% (2.3 to 28.7), *P* = 0.02. There were no other associations between the predictor covariates and time spent within, above and below range. Participants spent a lower proportion of time with SpO_2_ in target range during the night compared to the day; difference (95% CI) − 9.8% (− 4.7 to − 14.8), *P* < 0.001. Nocturnal data was included in 15 of the 17 participants with OSA/OHS; the mean (SD) proportion of time spent below range overnight in this group was 38.8% (34.5). Nocturnal data was included in 58 of the 63 participants without OSA/OHS; the proportion of time spent below range overnight in this group was 30.3% (31).

**Table 4:** Effect of predictor variables on percentage of time spent in, above and below range

Predictor variable	SpO_2_ below target range % increase or difference (95% CI)		SpO_2_ within target range % increase or difference (95% CI)		SpO_2_ above target range % increase or difference (95% CI)	
	Univariate	Multivariate	Univariate	Multivariate	Univariate	Multivariate
Age^a^	− 2.8 (− 7.1 to 1.6) *P* = 0.21	− 4.3 (− 8.7 to 0.4) *P* = 0.052	1.6 (− 2.5 to 5.7) *P* = 0.44	1.7 (− 2.3 to 5.7) *P* = 0.41	1.0 (− 3.2 to 5.2) *P* = 0.64	2.5 (− 1.3 to 6.3) *P* = 0.20
Oxygen adjustments^b^	1.5 (− 0.9 to 4.0) *P* = 0.22	2.7 (0.04 to 5.4) *P* = 0.047	0.5 (− 1.8 to 2.8) *P* = 0.69	− 1.0 (− 3.5 to 1.4) *P* = 0.40	− 2.1 (− 4.4 to 0.3) *P* = 0.08	− 1.7 (− 4.1 to 0.7) *P* = 0.15
HDU^c^	− 5.2 (− 18.3 to 7.9) *P* = 0.43	− 12.7 (− 27.1 to 1.7) *P* = 0.082	11.6 (− 0.5 to 23.7) *P* = 0.06	15.5 (2.3 to 28.7) *P* = 0.02	− 6.5 (− 19.1 to 6.0) *P* = 0.30	− 3.0 (− 15.6 to 9.7) *P* = 0.64
Target range^d^	7.9 (− 3.3 to 19.1) *P* = 0.16	8.4 (− 2.7 to 19.4) *P* = 0.13	12.8 (2.6 to 23.0) *P* = 0.014	13.1 (3.0 to 23.2) *P* = 0.012	− 21.0 (− 30.7 to − 11.3) *P* < 0.001	− 21.6 (− 31.4 to − 12.0) *P* < 0.001

^a^Per decade older

^b^Per one adjustment

^c^High dependency area versus non-high dependency area

^d^Target SpO_2_ range 92–96% versus reduced hypercapnic range

## Discussion

This study using continuous oximetry over a 24-h period demonstrated that acutely unwell medical patients receiving oxygen spent 56% of time with SpO_2_ within a prescribed target range. Patients with a reduced hypercapnic target range (88–92% or lower) spent 13% less time in range compared to patients with a target range of 92–96%, primarily due to a greater amount of time spent above range. Over a 24-h period this is equivalent to seven hours spent in which patients at risk of hypercapnic respiratory failure received excessive oxygen therapy. This was not fully accounted for by minor SpO_2_ fluctuations above range, as 16% of the total time spent above range was with SpO_2_ ≥ 5% above range. The 5% of time spent with profound desaturation to SpO_2_ < 80% also indicates these patients were also exposed to inadequate oxygen therapy.

This is concerning given the association between mortality and both excessive and inadequate oxygen administration to patients at risk of hypercapnic respiratory failure. In patients presenting with an acute exacerbation of COPD, administration of high-flow oxygen in the pre-hospital setting is associated with an increase in mortality [9]. A recent observational study of patients admitted to hospital with an acute exacerbation of COPD also demonstrated an association between over-oxygenation and risk of in-hospital adverse events [14]. Conversely, SpO_2_ < 88% is associated with an increased risk of serious adverse outcomes in patients presenting to an Emergency Department [15]. Recent evidence suggests a U-shaped association between SpO_2_ and mortality in this patient population, irrespective of the presence of hypercapnia, whereby risk of death increases even with modest deviations of SpO_2_ both above and below the target range of 88–92% [16, 17].

In the 92–96% group, insufficient oxygen therapy was a more common problem with 31% of time, or approximately seven and a half hours over a 24-h period spent below range. Again, this was not fully accounted for by minor fluctuations below range, as 18% of the total time spent below range was with SpO_2_ 87% or below. This is noteworthy given a previous study of inpatients in a general ward setting demonstrated a 2.4-fold increase in risk of in-hospital mortality relating to an oxygen saturation below 90% [10].

The histogram density plots demonstrate that not only was SpO_2_ frequently outside of the target range, but also that on an individual participant level, there were prolonged periods with SpO_2_ both significantly above and below range. This highlights the difficulty of achieving appropriate oxygen delivery based upon intermittent SpO_2_ measurement and manual oxygen titration in a ward setting. This method of oxygen titration has remained unchanged in clinical practice since the introduction of routine pulse oximetry. More recently, oxygen delivery systems have been developed which are able to automatically titrate the delivered oxygen concentration in order to achieve a target SpO_2_, based on continuous feedback from an associated sensor [18]. This method of automatic oxygen titration results in an increased proportion of time spent with SpO_2_ in target range compared to manual oxygen titration in a ward setting [19], the emergency department [20] and following major surgery [21]. However, it remains to be elucidated whether the use of such systems translates into improved clinical outcomes and what role they may play in routine clinical care.

There are no previous studies using continuous oximetry to determine time exposure to SpO_2_ outside of a prescribed target range, however our results are consistent with other work demonstrating over-oxygenation is common in a general ward setting. The BTS national emergency oxygen audit in 2015 demonstrated 21% of patients with a prescribed target range had SpO_2_ above this range when measured at a single timepoint [12]. A retrospective study of patients admitted to hospital with an acute exacerbation of COPD demonstrated over-oxygenation in 62% of admissions [14]. A further study using random pulse oximetry measurements in hospitalised patients receiving oxygen on a ward setting demonstrated oxygen was not required in 61% of cases [22]. The percentage of time spent in range is also in keeping with the control arm of studies comparing automated to manual oxygen titration in a ward setting, although these results were obtained in the context of a randomised controlled trial and may not represent usual clinical practice [19, 23]. Our finding that under-oxygenation is also common, particularly overnight, reflects the use of continuous SpO_2_ measurement over a 24-h period which detected desaturation which may otherwise go unnoticed between nursing observations. The novel aspects of this study include: the non-interventional use of continuous oximetry to accurately reflect the “real world” practice of manual oxygen titration in a ward setting, the evaluation of clinical factors which influence time spent in range and finally the detailed description of time exposure to significant deviation from a prescribed SpO_2_ target range.

The strengths of this study include the use of a small pulse oximeter with a concealed screen which minimised the chance of influencing clinical management. The inclusion of two study sites also increases the generalisability of the findings. We also only included SpO_2_ data with adequate signal quality for analysis, minimising the risk or artefactual SpO_2_ readings influencing the proportion of time outside of range. The inclusion of only medical patients is a limitation of the study and we cannot be sure the results would apply to other patient populations. Patients with OSA/OHS spent a greater proportion of time below range overnight compared to patients without OSA/OHS, although the difference was small. It is therefore possible some of the time spent below range was due to the presence of OSA/OHS. The study was conducted during the COVID-19 pandemic and it is likely that the effect of recent lockdowns and social distancing reduced hospital admissions due to respiratory viruses [24]. This may explain why only eight exacerbations of COPD were included in this study. For the proportion of time spent below range, we cannot exclude the possibility that non-compliance with oxygen therapy or lack of oxygen therapy when mobilising contributed to our findings, which may not reflect the efficacy of oxygen titration. It is possible that minor fluctuations in SpO_2_ above and below range may be due to discrepancy between SpO_2_ measured by the nursing staff which was used to titrate oxygen and SpO_2_ measured by the concealed research pulse oximeter. In addition, we did not include clinical outcomes in this study and cannot be sure of the clinical relevance of the findings.

## Conclusions

In conclusion, this study provides evidence that acutely unwell medical patients in a ward setting spend significant periods of time with SpO_2_ both above and below the prescribed target range while receiving oxygen. Further studies are required to determine the clinical impact this may have and whether the use of automatic oxygen titration in this setting can reduce patient exposure to over and under-oxygenation.

## Data Availability

The datasets used and/or analysed during the current study are available from the corresponding author on reasonable request.

## Abbreviations

SpO_2_: Peripheral oxygen saturation
SD: Standard deviation
CI: Confidence interval
BTS: British Thoracic Society
TSANZ: Thoracic Society of Australia and New Zealand
WRH: Wellington Regional Hospital
HVH: Hutt Valley Hospital
COPD: Chronic obstructive pulmonary disease
OSA: Obstructive sleep apnoea syndrome
OHS: Obesity hypoventilation syndrome
ICU: Intensive care unit
